# Extracellular Signal-Regulated Kinase 5 is Required for Low-Concentration H_2_O_2_-Induced Angiogenesis of Human Umbilical Vein Endothelial Cells

**DOI:** 10.1155/2017/6895730

**Published:** 2017-04-30

**Authors:** Shan Jiang, Dongxin Zhang, Hong Huang, Yonghong Lei, Yan Han, Weidong Han

**Affiliations:** ^1^Department of Plastic and Reconstructive Surgery, Chinese PLA General Hospital, Beijing 100853, China; ^2^Medicine School of Chinese PLA, Beijing 100853, China; ^3^Department of General Surgery, Beijing Tongren Hospital, Capital Medical University, Beijing 100730, China; ^4^Institute of Basic Medicine, Chinese PLA General Hospital, Beijing 100853, China; ^5^Medicine School of Nankai University, Tianjin 300071, China

## Abstract

*Background*. The aim of this study was to assess the effects of low concentrations of H_2_O_2_ on angiogenesis of human umbilical vein endothelial cells (HUVECs) in vitro and explore the underlying mechanisms.* Methods*. HUVECs were cultured and stimulated with different concentrations of H_2_O_2_. Flow cytometric analysis was used to select an optimal concentration of H_2_O_2_ for the following experiments. Cell proliferation, migration, and tubule formation were evaluated by Cell Counting Kit-8 (CCK-8) assays, scratch wound assays, and Matrigel tubule formation assays, respectively. For gain and loss of function studies, constitutively active MEK5 (CA-MEK5) and ERK5 shRNA lentiviruses were used to activate or knock down extracellular signal-regulated kinase 5 (ERK5).* Results*. We found that low concentrations of H_2_O_2_ promoted HUVECs proliferation, migration, and tubule formation. ERK5 in HUVECs was significantly activated by H_2_O_2_. Enhanced ERK5 activity significantly amplified the proangiogenic effects of H_2_O_2_; in contrast, ERK5 knock-down abrogated the effects of H_2_O_2_.* Conclusions*. Our results confirmed that low concentrations of H_2_O_2_ promoted HUVECs angiogenesis in vitro, and ERK5 is an essential mediator of this process. Therefore, ERK5 may be a potential therapeutic target for promoting angiogenesis and improving graft survival.

## 1. Introduction

Tissue transplantation is commonly used in plastic and reconstructive surgery. Timely and effective revascularization is important in ensuring graft survival, and angiogenesis plays an essential role in this process. Angiogenesis, the formation of new blood vessels from existing vasculature, involves many cellular components and signaling pathways, including reactive oxygen species (ROS), which play an important role in this process [1].

ROS, including hydrogen peroxide (H_2_O_2_), superoxide radicals (O^2−^), and hydroxyl radicals (OH^−^), are important signaling molecules that regulate multiple biological responses, including angiogenesis [2]. ROS, H_2_O_2_ in particular, have biphasic effects on angiogenesis; ROS at physiological levels mediate endothelial cell proliferation, migration, tubular formation, and enhanced angiogenesis, but ROS at pathological levels result in detrimental effects, including cell apoptosis, death, and impaired angiogenesis [3–5]. Low concentrations of ROS induce angiogenesis, but the signaling cascades linked to this outcome are unclear.

Among various ROS downstream signaling molecules, mitogen-activated protein kinases (MAPKs) have a crucial role [6]. The MAPK signaling pathway is highly conserved and involved in many different cellular functions, such as cell survival, proliferation, migration, and differentiation. To date, four different MAPK family members have been identified: p38, c-Jun-amino-terminal kinase (JUN), extracellular signal-regulated kinase 1/2 (ERK1/2), and ERK5 [7]. Among them, ERK5, which is also known as big MAP kinase 1 (BMK1), is the most recently identified family member [8]. ERK5 can be activated to a large extent by H_2_O_2_, suggesting that, compared with other MAPK family members, ERK5 is a redox-sensitive kinase [9]. Targeted deletion of ERK5 in mice revealed its crucial functions in angiogenesis [10].

Since both H_2_O_2_ and ERK5 play important roles in angiogenesis and ERK5 is redox-sensitive, we hypothesized that low-concentration H_2_O_2_ promotes endothelial cell angiogenesis through the ERK5 pathway. To verify this hypothesis, we investigated the role of ERK5 in H_2_O_2_-induced human umbilical vein endothelial cell (HUVECs) proliferation, migration, and tubule formation.

In this study, we found that ERK5 activation by H_2_O_2_ was critical for its proangiogenic function; downregulation of p-ERK5 by shERK5 inhibited H_2_O_2_ proangiogenic activity in vitro, whereas upregulation of p-ERK5 by CA-MEK5 facilitated H_2_O_2_ proangiogenic action. The concentration of H_2_O_2_ (50 *μ*mol/L) used in our study was in the range of that reached under pathophysiological conditions in vivo (from 0.2 nmol/L in red blood cells to 200 *μ*mol/L in wound fluid) [11, 12]; therefore, the findings of the present study may be applicable to clinical settings.

## 2. Methods

### 2.1. Cell Culture

HUVECs were acquired from China Infrastructure of Cell Line Resources (Beijing, China). Cells were grown in Dulbecco's modified Eagle's medium (DMEM, HyClone, USA) containing 10% fetal bovine serum (FBS, HyClone, USA) and 1% penicillin-streptomycin (Gibco, USA). The cell cultures were incubated at 37°C in a humidified atmosphere with 5% CO_2_. Cells were used for experiments at 80–90% confluency after 2-3 days of culture. In some cases, 5 U/ml catalase (Sigma, USA) was added 5 min before H_2_O_2_ was administered.

### 2.2. Gain and Loss of Function Studies

For gain of function studies, a constitutively active MEK5 (CA-MEK5) lentivirus was used to activate ERK5. A nonspecific green fluorescent protein (GFP) lentivirus with the same multiplicity of infection (MOI, 20) was used as a negative control. For loss of function studies, ERK5 shRNA (shERK5) lentivirus was used to reduce ERK5 expression in endothelial cells. A nonsilencing shRNA lentivirus with the same MOI was used as a negative control (control siRNA). Cells were seeded in 6-well plates, cultured overnight, and then incubated with lentivirus for 48 h for infection. All the plasmids and lentiviruses used were constructed by the Shanghai Obio Technology Company (Shanghai, China).

### 2.3. Detection of Apoptosis

After a 30 min incubation with different concentrations of H_2_O_2_, cells were labeled with phycoerythrin- (PE-) conjugated annexin V and 7-amino-actinomycin (7-AAD) (BD Pharmingen, USA) following the kit instructions. Approximately 5,000 cells were counted from each sample using flow cytometry (BD Biosciences, USA), and early apoptotic cells were defined as annexin V positive/7-AAD negative.

### 2.4. Western Blot Analysis

Cells were lysed in RIPA buffer (50 mmol/L Tris-HCl, 150 mmol/L NaCl, 1% NP-40, 0.5% sodium deoxycholate, and 0.1% SDS) containing a protease inhibitor cocktail (Roche, USA). The protein concentration was measured using a BCA kit, and 30 *μ*g of protein per sample was used for polyacrylamide gel electrophoresis and then transferred to nitrocellulose membranes. The membranes were blocked with fat-free milk or 5% BSA for 1 h at room temperature and then incubated with various antibodies: ERK5 (#3372, Cell Signaling Technology, 1 : 1000), phosphorylated-ERK5 (#07-507, Millipore, 1 : 1000), MEF2C (#5030T, Cell Signaling Technology, 1 : 1000), phosphorylated-MEF2C (sc-377535, Santa Cruz, 1 : 200), ERK1/2 (#9102, Cell Signaling Technology, 1 : 1000), p-ERK1/2 (#9101, Cell Signaling Technology, 1 : 1000), and *β*-actin (ab8226, Abcam, 1 : 1000) overnight at 4°C. After the membranes were washed with TBST, they were incubated with HRP-linked secondary antibodies: goat anti-rabbit IgG (#7074, Cell Signaling Technology, 1 : 2000) or goat anti-mouse IgG (ab6728, Abcam, 1 : 2000) for 1 h at room temperature. The density of the protein bands was determined by ImageJ software. Densitometric values of the ERK5, MEF2C, and ERK1/2 bands were normalized to that of *β*-actin. Phosphorylated protein levels were normalized to the respective total protein levels.

### 2.5. Detection of Cell Proliferation

Cell proliferation was assessed using a Cell Counting Kit-8 (CCK-8, Dojindo, Japan) assay. Following the manufacturer's instructions, HUVECs were seeded in 96-well plates at a density of 1 × 10^3^ cells per well. After the cells were cultured in a standard environment for 24 h, they were analyzed using CCK-8 assays. Following treatment with PBS, H_2_O_2_ (50 *μ*mol/L), or catalase (5 U/ml) and H_2_O_2_ (50 *μ*mol/L) for 30 min, the culture media were changed to normal medium. Then, 10 *μ*l CCK-8 was added, and cells were further cultured for 2.5 h. The optical density (OD) at 450 nm was measured using a microplate reader (Beijing Xinfeng Mechanical and Electrical Technology Co., China).

### 2.6. Cell Migration Assay

Cell migration was assessed using scratch wound assays. HUVECs were seeded in 6-well plates at a density of 1 × 10^6^ cells per well (confluence) and cultured overnight. Following treatment with PBS, H_2_O_2_ (50 *μ*mol/L), or catalase (5 U/ml) and H_2_O_2_ (50 *μ*mol/L) for 30 min, a scratch was made by a sterile 200 *μ*l pipette tip in the center of each well and washed twice with PBS. The wounded HUVECs monolayers were then incubated with medium containing 2% FBS for 12 h or 24 h. Photographs were taken at 0 h and 12 h or 24 h at fixed locations along the scratch with an Olympus microscope, and the closure of the wound area was quantified using ImageJ software.

### 2.7. Tubule Formation Assay

In vitro tubule formation of HUVECs was assayed on Matrigel (Corning, USA). Briefly, Matrigel (400 *μ*l per well) was coated on 6-well plates and incubated at 37°C for 30 min to form a solid gel. Cells were pretreated with PBS, H_2_O_2_ (50 *μ*mol/L), or catalase (5 U/ml) and H_2_O_2_ (50 *μ*mol/L) for 30 min and then trypsinized, collected, and seeded onto Matrigel-coated wells (1 × 10^4^ cells per well) in complete medium (DMEM containing 10% FBS and 1% penicillin-streptomycin) and cultured for 4 to 8 h. Tubule formation was observed with an Olympus microscope and analyzed by measuring branch length and counting tubule number with ImageJ software.

### 2.8. Enzyme Linked Immunosorbent Assay (ELISA)

Cells were seeded in 60 mm tissue culture dishes at a density of 1 × 10^6^ cells per well and cultured overnight in normal medium. Following treatment with PBS or 50 *μ*mol/L H_2_O_2_ for 30 min, the medium was changed to fresh normal medium. After 12 h or 24 h culture, the cell supernatant was collected, and the concentration of human vascular endothelial growth factor (VEGF) protein was determined by a human VEGF ELISA kit (R&D Systems, USA) according to the manufacturer's instructions.

### 2.9. Statistical Analysis

All data are presented as the mean ± standard deviation (SD). Comparison among multiple groups was performed by one-way analysis of variance (ANOVA) followed by the SNK post hoc test. The results were considered statistically significant when the *p* value was less than 0.05 (*p* < 0.05). Each experiment included triplicate measurements for each condition tested, and the experiment was repeated at least three times.

## 3. Results

### 3.1. Effects of Different Concentrations of H_2_O_2_ on HUVEC Viability

**Figure 1:**
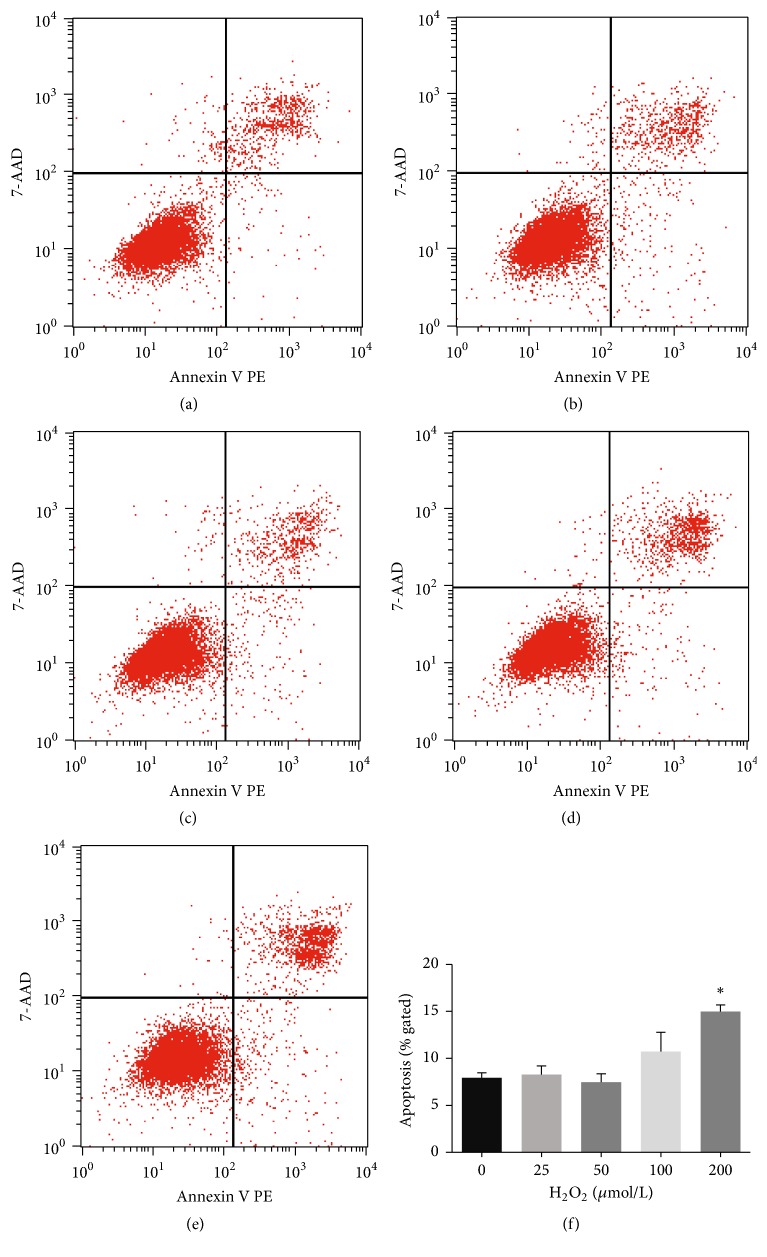
Effects of different concentrations of H_2_O_2_ on HUVEC viability. (a–e) Scatter plots of flow cytometric results with annexin V (horizontal axis) and 7-AAD (vertical axis). Cells were labeled with annexin V and 7-AAD after a 30 min incubation with different concentrations of H_2_O_2_. The conditions were as follows: (a) 0 *μ*mol/L H_2_O_2_ (control), (b) 25 *μ*mol/L H_2_O_2_, (c) 50 *μ*mol/L H_2_O_2_, (d) 100 *μ*mol/L H_2_O_2_, and (e) 200 *μ*mol/L H_2_O_2_. Early apoptotic cells were defined as annexin V positive/7-AAD negative. (f) Quantitative analysis of cell apoptosis with different concentrations of H_2_O_2_ (mean ± SD of six separate experiments, ^*∗*^*p* < 0.05 versus control, and one-way ANOVA followed by SNK post hoc test).

To determine the optimal concentration of H_2_O_2_, we assessed the effects of five different concentrations. The results showed that 0–50 *μ*mol/L H_2_O_2_ did not induce significant apoptosis of HUVECs. However, 100 *μ*mol/L H_2_O_2_ increased the apoptotic rate of the cells, and 200 *μ*mol/L H_2_O_2_ induced significant cell apoptosis (Figure 1). Based on these results and proangiogenic preexperiment results that proangiogenic effect was more significant with 50 umol/L H_2_O_2_ (versus control group) than with 25 umol/L H_2_O_2_, we used 50 *μ*mol/L H_2_O_2_ in the following experiments.

### 3.2. Low Levels of H_2_O_2_ Promote HUVEC Proliferation, Migration, and Tubule Formation

**Figure 2:**
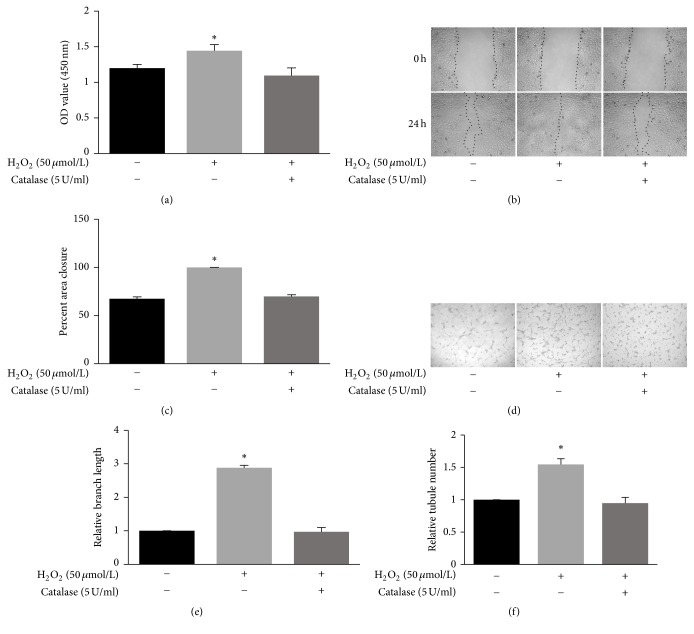
Low concentrations of H_2_O_2_ promote HUVEC proliferation, migration, and tubule formation. (a) Cell proliferation assessed by CCK-8 assays. HUVECs were treated with PBS, H_2_O_2_ (50 *μ*mol/L), or catalase (5 U/ml) and H_2_O_2_ (50 *μ*mol/L) for 30 min. Then, 10 *μ*l CCK-8 was added. The OD at 450 nm was measured 2.5 h later (mean ± SD of six separate experiments, ^*∗*^*p* < 0.05 versus control, and one-way ANOVA followed by SNK post hoc test). (b, c) Cell migration assessed by scratch wound assays. HUVECs were seeded at confluence and cultured overnight. Following treatment with PBS, H_2_O_2_ (50 *μ*mol/L), or catalase (5 U/ml) and H_2_O_2_ (50 *μ*mol/L) for 30 min, a scratch was made. The wounded HUVECs monolayers were then incubated with medium containing 2% FBS for 24 h. Photographs were taken at 0 h and 24 h at fixed locations along the scratch, and the closure of the wound area was quantified (mean ± SD of six separate experiments, ^*∗*^*p* < 0.05 versus control, and one-way ANOVA followed by SNK post hoc test). (d, e, f) In vitro tubule formation. Tubule formation was assayed on Matrigel. Cells were pretreated with PBS, H_2_O_2_ (50 *μ*mol/L), or catalase (5 U/ml) and H_2_O_2_ (50 *μ*mol/L) for 30 min and then seeded onto Matrigel-coated wells in normal medium and cultured for 4 to 8 h. Tubule formation was analyzed by measuring branch length and counting tubule number (mean ± SD of six separate experiments, ^*∗*^*p* < 0.05 versus control, and one-way ANOVA followed by SNK post hoc test).

Stimulation of endothelial cell proliferation is essential for angiogenesis. As shown in Figure 2(a), treatment with 50 *μ*mol/L H_2_O_2_ significantly increased HUVECs proliferation, and this effect was completely abrogated by catalase, an enzyme that eliminates H_2_O_2_. Additionally, cell migration is an important step in angiogenesis. Scratch wound assays were performed; the area recovered represents endothelial cell migration (Figure 2(b)). Control cells recovered (67 ± 2.5)% after 24 h. Cells treated with 50 *μ*mol/L H_2_O_2_ increased migration to 100%. The cells treated with 50 *μ*mol/L H_2_O_2_ and catalase (5 U/ml) did not differ from the control cells [(70 ± 1.9)%, Figure 2(c)]. Furthermore, tubule formation is one of the hallmarks of angiogenesis, along with cell proliferation and migration. To assess the effect of low-concentration H_2_O_2_ on endothelial cell angiogenesis, we performed a Matrigel tubule formation assay (Figure 2(d)). After the cells were plated on Matrigel, control cells formed few capillary-like tubes, while cells treated with 50 *μ*mol/L H_2_O_2_ formed more capillary-like tube structures (branch length: 2.9-fold increase; tubule number: 1.5-fold increase). As observed previously, cells treated with 50 *μ*mol/L H_2_O_2_ and 5 U/ml catalase did not differ from those of the control. Overall, low-concentration H_2_O_2_ promoted HUVECs angiogenesis; however, catalase eliminated all H_2_O_2_ proangiogenic effects.

### 3.3. Low Concentrations of H_2_O_2_ Induce ERK5 Activation in HUVECs

**Figure 3:**
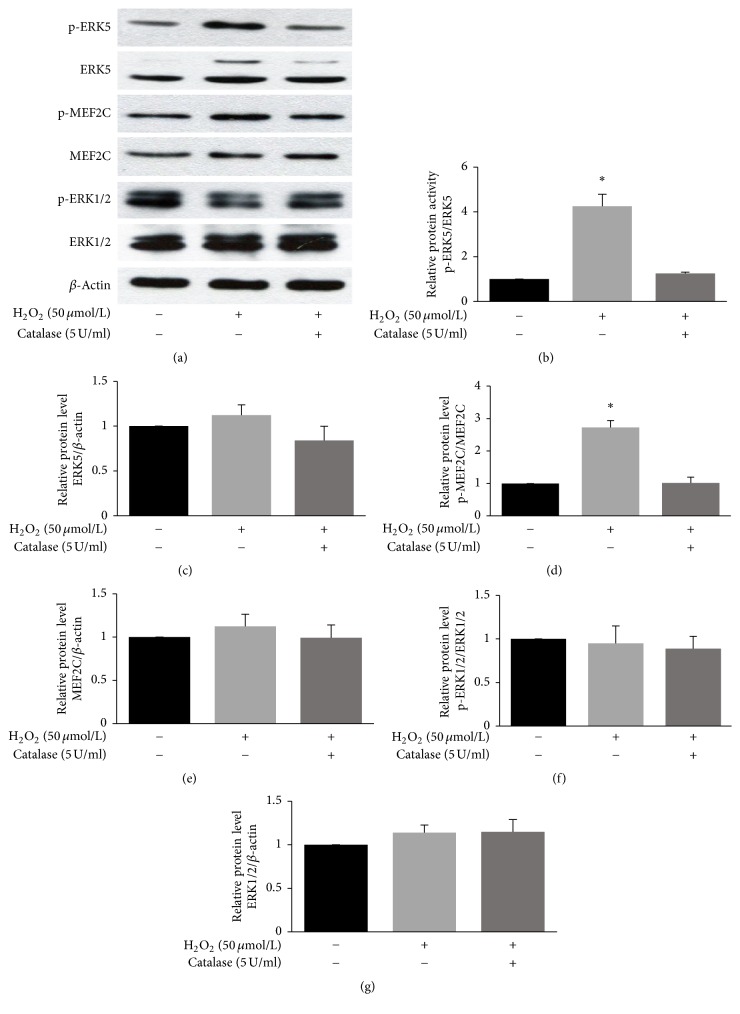
Low-concentration H_2_O_2_ induced ERK5 activation in HUVECs. (a) ERK5, p-ERK5, MEF2C, p-MEF2C, ERK1/2, and p-ERK1/2 protein levels were measured by western blotting. Following treatment with PBS, H_2_O_2_ (50 *μ*mol/L), or catalase (5 U/ml) and H_2_O_2_ (50 *μ*mol/L) for 30 min, cells were lysed, and the lysates were resolved by 10% or 6% SDS-PAGE. (b, c, d, e, f, g) Densitometric quantification of ERK5, p-ERK5, MEF2C, p-MEF2C, ERK1/2, and p-ERK1/2 expression (mean ± SD of three separate experiments, ^*∗*^*p* < 0.05 versus control, and one-way ANOVA followed by SNK post hoc test).

We examined whether ERK5 was activated by low-concentration H_2_O_2_ in HUVECs. Endogenous ERK5 activity was measured by assessing phosphorylation of ERK5 and its substrate, MEF2C. H_2_O_2_ treatment of the cells resulted in activation of ERK5 without changes in total ERK5. Pretreatment of the cells with 5 U/ml catalase inhibited ERK5 activation by H_2_O_2_. ERK1/2 and p-ERK1/2 protein levels did not change significantly among the experimental groups (Figure 3).

### 3.4. ERK5 Mediates Low-Concentration H_2_O_2_-Induced HUVEC Angiogenesis In Vitro

**Figure 4:**
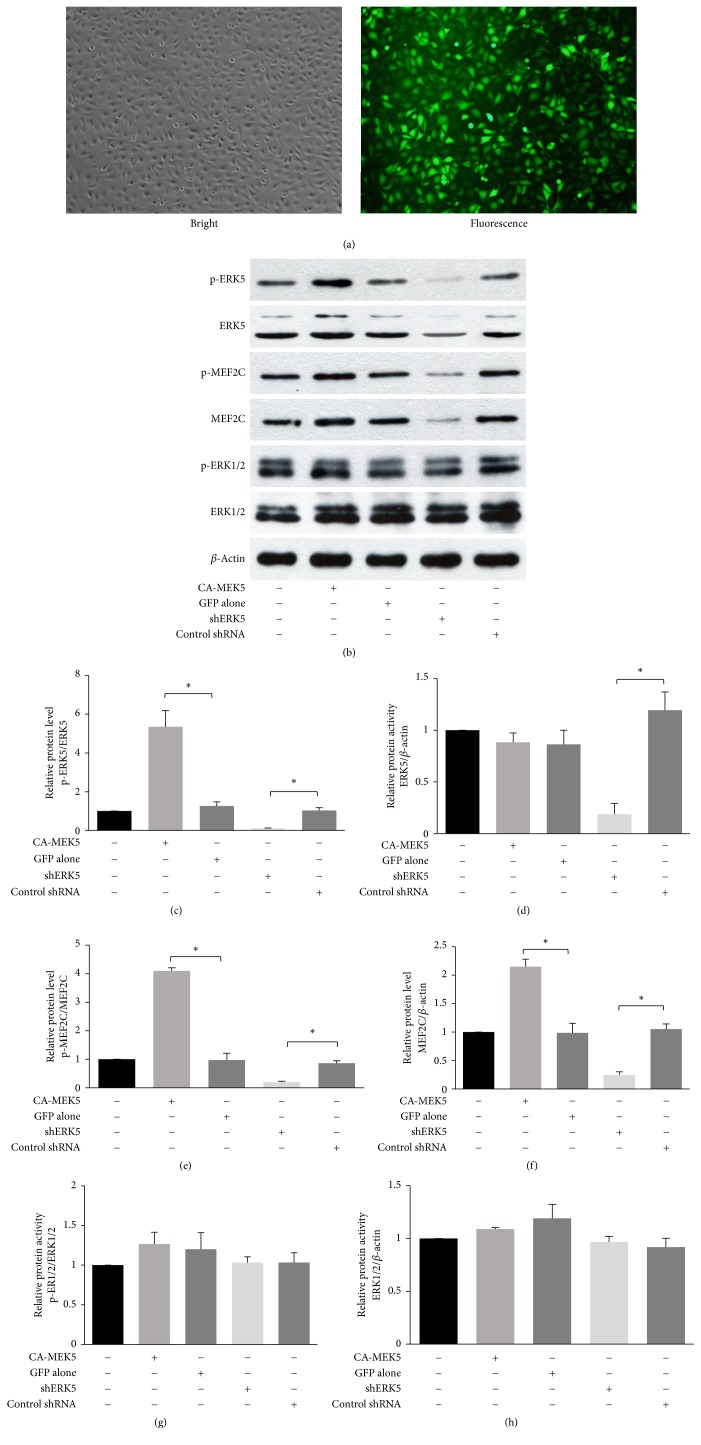
Construction of high or low p-ERK5 HUVEC populations. (a) Images of lentivirus-transfected HUVECs. (b) ERK5, p-ERK5, MEF2C, p-MEF2C, ERK1/2, and p-ERK1/2 protein levels were determined by western blotting. Different cell populations were lysed, and the lysates were resolved by 10% or 6% SDS-PAGE. (c, d, e, f, g, h) Densitometric quantification of ERK5, p-ERK5, MEF2C, p-MEF2C, ERK1/2, and p-ERK1/2 expression (mean ± SD of three separate experiments, ^*∗*^*p* < 0.05, and one-way ANOVA followed by SNK post hoc test).

To determine whether ERK5 was essential for H_2_O_2_-induced angiogenesis, we generated HUVECs populations with CA-MEK5 expression or Erk5 knock-down and their respective control populations. Lentiviral transfection efficiency was greater than 90%, as assessed by GFP expression (Figure 4(a)), and therefore, cell sorting or selection was not necessary. As shown in Figures 4(b)–4(f), transfection with CA-MEK5 strongly activated ERK5, and the ERK5 knock-down efficiency by a specific shRNA was 80%. No significant change of ERK1/2 and p-ERK1/2 protein levels was seen in all cell populations (Figures 4(g)–4(h)). The resulting cells were tested in the following in vitro angiogenesis assays.

**Figure 5:**
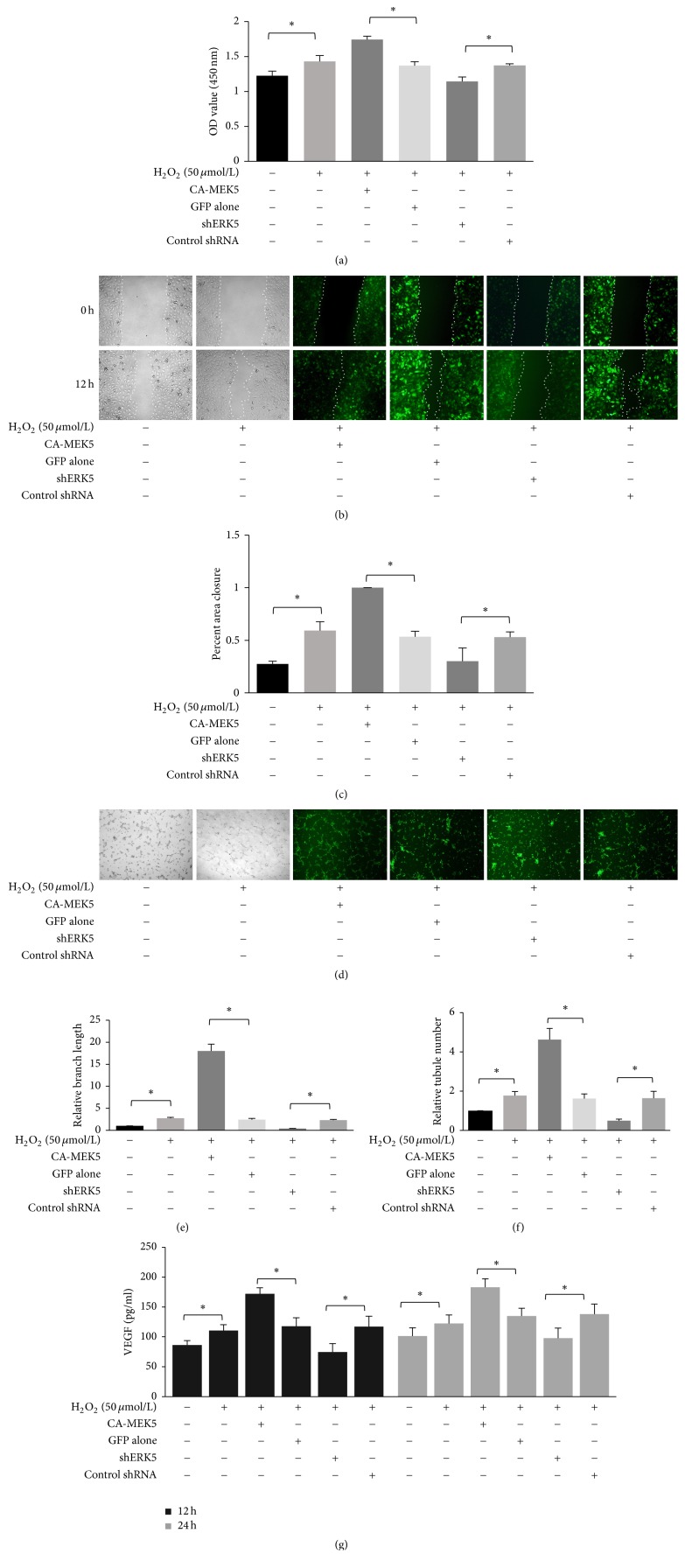
Activated ERK5 is essential for low-concentration H_2_O_2_-induced HUVEC angiogenesis. (a) Cell proliferation assessed by CCK-8 assays. Cells were treated with PBS or H_2_O_2_ (50 *μ*mol/L) for 30 min. Then, 10 *μ*l CCK-8 was added. The OD value at 450 nm was measured 2.5 h later (mean ± SD of six separate experiments, ^*∗*^*p* < 0.05, and one-way ANOVA followed by SNK post hoc test). (b, c) Cell migration assessed by scratch wound assays. Cells were seeded at confluence and cultured overnight. Following treatment with PBS or H_2_O_2_ (50 *μ*mol/L) for 30 min, a scratch was made. The wounded cell monolayers were then incubated with medium containing 2% FBS for 12 h. Photographs were taken at 0 h and 12 h at fixed locations along the scratch, and closure of the wound was quantified (mean ± SD of six separate experiments, ^*∗*^*p* < 0.05, and one-way ANOVA followed by SNK post hoc test). (d, e, f) In vitro tubule formation. Tubule formation was assayed on Matrigel. Cells were pretreated with PBS or H_2_O_2_ (50 *μ*mol/L) for 30 min and then seeded onto Matrigel-coated wells in normal medium and cultured for 4 to 8 h. Tubule formation was analyzed by measuring branch length and counting tubule number (mean ± SD of six separate experiments, ^*∗*^*p* < 0.05, and one-way ANOVA followed by SNK post hoc test). (g) VEGF secretion assessed by ELISA. Cells were treated with PBS or H_2_O_2_ (50 *μ*mol/L) for 30 min; then the medium was changed to fresh normal medium. After 12 h or 24 h culture, the cell supernatant was collected, and the concentration of VEGF was determined by a human VEGF ELISA kit (mean ± SD of six separate experiments, ^*∗*^*p* < 0.05, and one-way ANOVA followed by SNK post hoc test).

In HUVECs transfected with CA-MEK5, cell proliferation (Figure 5(a)), migration (Figures 5(b) and 5(c)), and tubule formation (Figures 5(d), 5(e), and 5(f)) by H_2_O_2_ were significantly amplified. In contrast, ERK5 knock-down abrogated the proangiogenic effects of low concentrations of H_2_O_2_. Furthermore, VEGF secretion stimulated by H_2_O_2_ was also significantly amplified in HUVECs transfected with CA-MEK5, while VEGF level decreased in HUVECs transfected with ERK5 shRNA (Figure 5(g)), These results suggest that low concentrations of H_2_O_2_ induced HUVEC angiogenesis through the ERK5 signaling pathway.

## 4. Discussion

Angiogenesis is a complex, multifaceted process that requires the coordinated proliferation, migration, and ultimate differentiation of endothelial cells as well as other cells to form a lumen-containing vessel capable of allowing blood flow. This study is the first to reveal that ERK5 is specifically required for low-concentration H_2_O_2_-induced angiogenesis in HUVECs. H_2_O_2_ (50 *μ*mol/L) promoted HUVEC proliferation, migration, and tubule formation (Figure 2); furthermore, gain/loss of function studies revealed that ERK5 mediated these processes (Figure 5). A series of studies of transgenic ERK5 mice showed that ERK5 plays a critical role in endothelial cell function. ERK5-null mice died of cardiovascular defects and impaired angiogenesis on embryonic day 10.5 [13–15]. Additionally, the conditional ERK5 knock-out adult mouse died within 2-3 weeks due to endothelial failure [16]. Furthermore, ERK5 was shown to be essential in protecting endothelial cells from apoptosis induced by serum deprivation and tumor necrosis factor [17]. The present study further supports the key role of ERK5 in endothelial cell function.

ROS, especially H_2_O_2_, have been reported to have biphasic effects on angiogenesis—at physiological levels, they promote angiogenesis, but excess levels impair angiogenesis. The tolerance of cells to H_2_O_2_ depends on the cell type and cellular context [18–20]. As shown by our results, 50 *μ*mol/L H_2_O_2_ was not toxic to HUVECs and did not cause significant apoptosis. However, 200 *μ*mol/L H_2_O_2_ induced significant cell apoptosis (Figure 1). High levels of H_2_O_2_ may trigger other signaling pathways that lead to cell apoptosis or death, and ERK5 activation could not overcome these oxidative insults.

Previous studies have shown that a wide variety of mitogens, including stress stimuli such as H_2_O_2_, can activate the ERK5 signaling pathway, as well as the other MAP kinases. The present findings that H_2_O_2_ stimulated significant activation of ERK5 (Figure 3) are consistent with a previous report showing that H_2_O_2_ activated ERK5 in vascular smooth muscle cells [21]. In addition, H_2_O_2_-induced activation of ERK5 in cultured fibroblasts was also observed [22]. Therefore, activation of ERK5 by H_2_O_2_ may be common in many cell types. The intracellular signaling mechanisms that lead to ERK5 activation have been investigated. A specific ERK5 upstream kinase, MEK5, and c-Src tyrosine kinase have both been shown to mediate ERK5 activation in response to oxidative stress. However, whether low concentrations of H_2_O_2_ activate ERK5 through the same pathway requires further investigation.

The expression of ERK5 in different tissues appears to be ubiquitous. Among several tissue-specific ERK5-knock-out mouse models, the endothelial ERK5-knock-out mouse showed an abnormal phenotype, indicating that ERK5 is critical for endothelial cell physiology [23]. ERK5 gain and loss of function experiments showed that ERK5 is required for low-concentration H_2_O_2_-induced angiogenesis.

shRNA-mediated knock-down of ERK5 or overexpression of CA-MEK5 significantly affected H_2_O_2_-mediated HUVECs proliferation. These findings are consistent with previous studies showing that ERK5 plays an important role in regulating cell cycle entry [24, 25]. The role of ERK5 in regulating cell proliferation remains unclear. Although previous studies have shown that proliferation of hematopoietic cells mediated by granulocyte-macrophage colony-stimulating factor (GM-CSF) [26], proliferation of vascular smooth muscle cells mediated by platelet-derived growth factor (PDGF) [27], and proliferation of neural stem/progenitor cells mediated by epidermal growth factor (EGF) [28] are all ERK5-dependent, many other studies using cells from Erk5^−/−^ and Mek5^−/−^ mice have shown that ERK5 and MEK5 are not required for cell cycle progression [16, 29]. Notably, Roberts et al. [30] found that ERK5 is dispensable for cellular proliferation in primary human microvascular endothelial cells seeded on a gelatin matrix. These contrasting results indicate that the effect of ERK5 on cell proliferation may depend on the stimuli, the cell type, and even the cellular context [31].

The finding that ERK5 activation also promoted endothelial cell migration suggests that ERK5 may be particularly important in the initiation of angiogenesis. Our results are consistent with those of previous reports [32–34]. In contrast, Spiering et al. [35] found that enhanced ERK5 signaling strongly inhibits endothelial cell migration and results in substantial morphological changes due to decreased focal contact turnover. Another recent report implicated ERK5 in Kruppel-like transcription factor (KLF2)/p21-activated kinase 1- (PAK1-) mediated inhibition of cell migration [36]. The explanation for these different findings is not clear. Various sources of cells and culture conditions may in part be responsible for this discrepancy.

Activation of ERK5 has previously been shown to regulate myoblast differentiation [37] by controlling the promyogenic actions of insulin-like growth factor 2 (IGF-2) [38]. Together with our results showing that ERK5 also mediates HUVECs tubule formation, these findings indicate that ERK5 regulates the differentiation of multiple cell types.

VEGF is a potent proangiogenic factor and activates ERK5 in both human and mouse endothelial cells [16]. However, whether ERK5 affects VEGF expression is unclear. A recent study indicated that the ERK5 pathway alters circulating VEGF level [39]. Our findings that 50 *μ*mol/L H_2_O_2_ stimulation significantly elevated VEGF secretion in cells transfected with CA-MEK5 and VEGF level decreased in ERK5 knock-down cells (Figure 5(g)) add further support to this statement and our conclusion.

Based on the results of the present study and several published studies, we hypothesize that ERK5 may be essential in the initial sprouting stages until the final differentiation stages of angiogenesis.

Low concentrations of H_2_O_2_ stimulate angiogenesis, indicating that pharmacologically regulating cellular ROS (H_2_O_2_ in particular) levels rationally may be an angiogenic strategy.

Furthermore, elucidating the in-depth mechanisms underlying H_2_O_2_-induced angiogenesis would help develop novel treatment strategies for promoting revascularization of grafts and improving their survival. The finding that ERK5 activity regulates low-concentration H_2_O_2_-induced angiogenesis suggests that ERK5 is a potential target for therapies that modulate graft survival. Many drugs have been developed to promote angiogenesis and improve graft survival after tissue transplantation. However, most of these drugs target growth factors (e.g., VEGF; fibroblast growth factor, FGF), endothelial-specific receptor tyrosine kinases, and other extracellular molecules [40]. Drugs that target intracellular signaling pathways, which are important for endothelial cell growth, migration, and differentiation, are very rare. The major reason may be that these pathways are critical in both endothelial cells and other types of cells. Notably, specific knock-down or knock-out of ERK5 in mice in several different cell types, such as cardiomyocytes, neurons, and mammary epithelial cells, all resulted in normal phenotypes without any obvious adverse effects. The lifespan of the aforementioned genetically modified mice was the same as that of their control littermates [23]. Therefore, endothelial cells may be the primary cell type affected by the loss of ERK5. Based on the above findings, pharmacological activators targeting the ERK5 pathway in vivo would predominantly affect endothelial cells, especially during angiogenesis, with little effect on other types of cells [41]. Additionally, it will be important in the future to identify the ERK5 substrates that mediate the proangiogenic effects of low concentrations of H_2_O_2_ in endothelial cells.

## 5. Conclusions

Here, we demonstrated a novel mechanism of H_2_O_2_-induced angiogenesis in HUVECs. We showed that low concentrations of H_2_O_2_ activated ERK5 in the HUVECs. Enhanced activity of ERK5 amplified the proangiogenic effects of H_2_O_2_. In contrast, reduced activity of ERK5 abolished the effects of H_2_O_2_. However, further experiments are needed to clarify the mechanisms involved. Nevertheless, we believe that the present study provides helpful insights into the role of ERK5 in angiogenesis and thus provides a theoretical basis for developing new treatment strategies to promote revascularization of grafts and improve their survival.
